# Supplementary material for: Dynamics of Dual Specificity Phosphatases and Their Interplay with Protein Kinases in Immune Signaling
Source: Int J Mol Sci. 2019 Apr 27;20(9):2086. doi: 10.3390/ijms20092086 (PMC6539644; doi:10.3390/ijms20092086)

Supplementary Tables

Supplementary Table S1. Expression of kinases and DUSPs (z-scores) in hematopoietic cells (matrix from Rieckmann et al, Nat Immunol (2017))

Supplementary Table S2. Matrix for corresponding RNA and protein expression data (z-scores) for T4 naïve, T4 TCM, B memory and classical monocyte cells

Supplementary Table S3. Expression of DUSPs and protein kinases in primary and secondary lymphoid tissue expression datasets.

Supplementary Table S4. Correlation matrix between DUSP and protein kinase expression in hematopoietic cells (data from Rieckmann et al, Nat Immunol (2017)). Spearman's rank correlation coefficient was used.

Supplementary Table S5. List of nodes and their properties from the comPPI interactome for DUSP family members. The interactions were obtained from the comPPI database and the network properties analyzed using Network Analyzer in Cytoscape.

Supplementary Table S6. Expression of kinases and DUSPs in activated hematopoietic cells (matrix from Rieckmann et al, Nat Immunol (2017)). Ratios obtained by dividing expression values in the activated state by expression values in the steady-state.

Supplementary Table S7. log2(fold-change) of kinases and DUSPs in LPS-stimulated hematopoietic cells. Ratios obtained by dividing expression values in the activated state by expression values in the steady-state and log transformation to the base 2. The study code indicated in the header can be referred to in Supplementary Table 1. The kind of biomolecule assayed and the time point after LPS stimulation is mentioned in the header.

Supplementary Table S8. List of DUSPs and kinases differentially expressed in monocytes and dendritic cells in response to LPS

Supplementary Table S9. List of enriched pathways containing proteins differentially expressed in monocytes and dendritic cells in response to LPS

Supplementary Table S10. Correlation matrix between DUSP and protein kinase expression in activated DCs and MOs. Spearman's rank correlation coefficient was used.

Supplementary Table S11. List of datasets used for the study

Supplementary Table S12. List of DUSP family genes used for the study and their details

Supplementary Table S13. List of protein kinases used for the study and their details

Supplementary Figures

Supplementary Figure 1. Baseline expression of A.DUSPs and B. protein kinases in hematopoietic cell line proteomic data. The raw hematopoietic cell expression data obtained from Rieckmann *et al*, *Nat Immunol* (2017) was scaled to obtain Z-scores. Z-scores were plotted as a heatmap using Morpheus and hierarchical clustering was carried out using Euclidean distance, complete linkage by both rows and columns. TUBB gene, observed to occur consistently across all cell types, was added to the heatmaps for comparison purpose

Supplementary Figure 2. DUSP interaction network. Protein-protein interaction data between DUSP and other proteins were obtained from Compartmentalized Protein-Protein Interaction (comPPI) Database were analyzed in Cytoscape using Network Analyzer to obtain network properties including Betweenness Centrality. Proteins with high Betweenness Centrality indicate primary regulatory proteins associated with DUSPs. Manual clustering of proteins within the vicinity of DUSPs was carried out using AutoAnnotate 1.3 in Cytoscape.

Supplementary Figure 3. (a) Differential expression of DUSPs in various activated hematopoietic cells. (b). Differential expression of protein kinases in various activated hematopoietic cells.

Supplementary Figure 4. Expression of (a) DUSPs and (b) protein kinases in activated hematopoietic cells. Expression ratios from activated and steady-state hematopoietic cells were calculated from expression data obtained from Rieckmann *et al*, *Nat Immunol* (2017) and log transformed. Expression values were plotted as a heatmap using Morpheus and hierarchical clustered by Euclidean distance and complete linkage by both rows and columns. **Similarity matrices for (c) DUSP and (d) protein kinase expression in hematopoietic cells.** DUSP and protein kinase expression in various cell types was correlated via Spearman's rank correlation.

Supplementary Figure 5: Venn diagrams showing the overlap of molecules differentially expressed in response to LPS in **(a).** murine and human dendritic cells (DCs). **(b)**. human dendritic cells and monocytes (upregulated molecules). **(c)** human dendritic cells and monocytes (downregulated molecules). **(d) Correlation of protein kinase and DUSP expression patterns in activated DCs and MOs**. The correlation was carried out using Spearman's rank correlation method to identify kinase-DUSP pairs that may have reciprocal activities.

Supplementary Figure 6. Network analysis of DUSPs and kinases that were (a). upregulated and (b). downregulated in activated murine dendritic cells

Supplementary Figure 7: Network analysis of DUSPs and kinases that were (a). upregulated and (b) downregulated in activated human dendritic cells

Supplementary Figure 8: Network analysis of DUSPs and kinases that were (a). upregulated and (b) downregulated in activated human monocytes

Supplementary figure 1

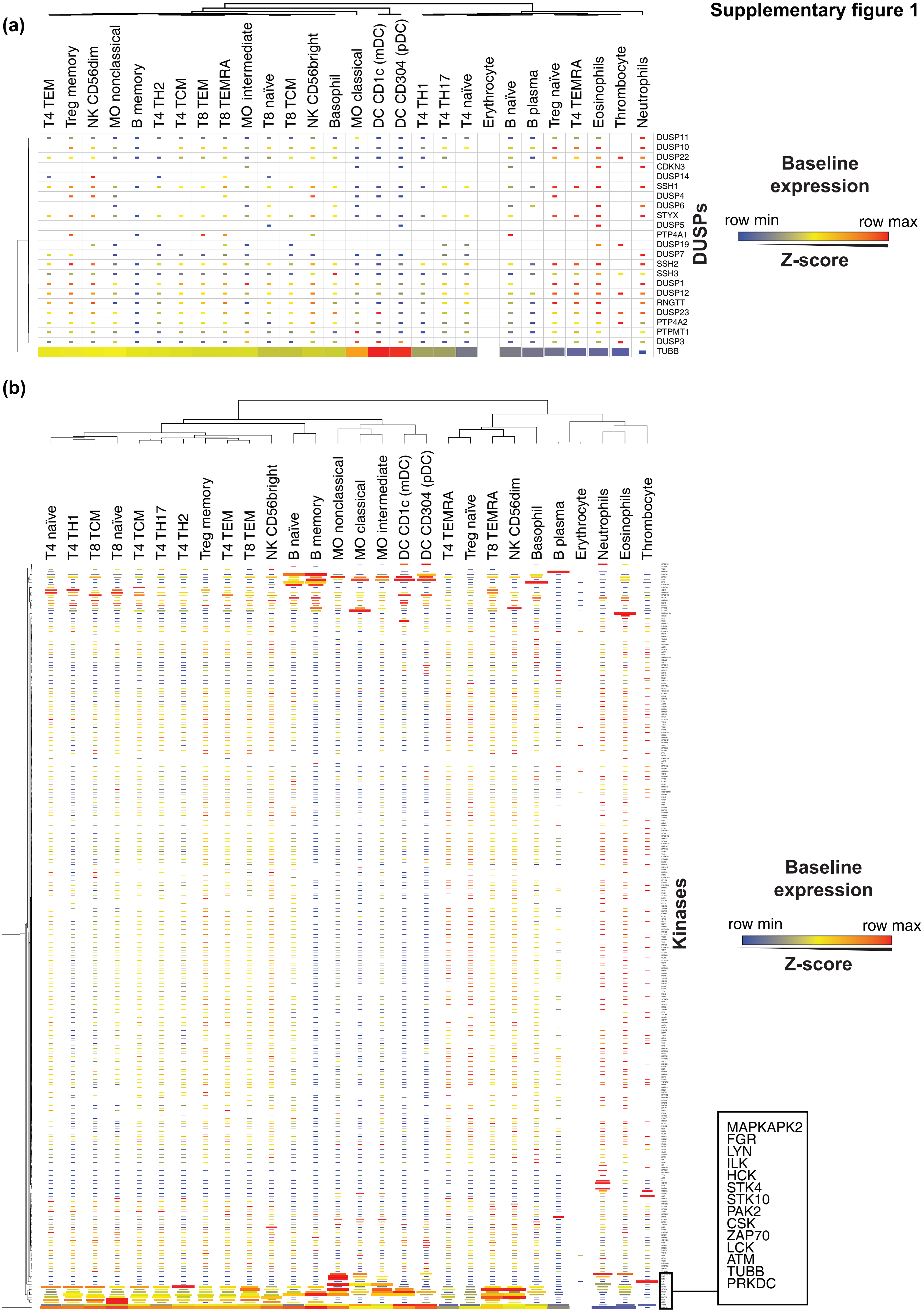

Supplementary figure 2

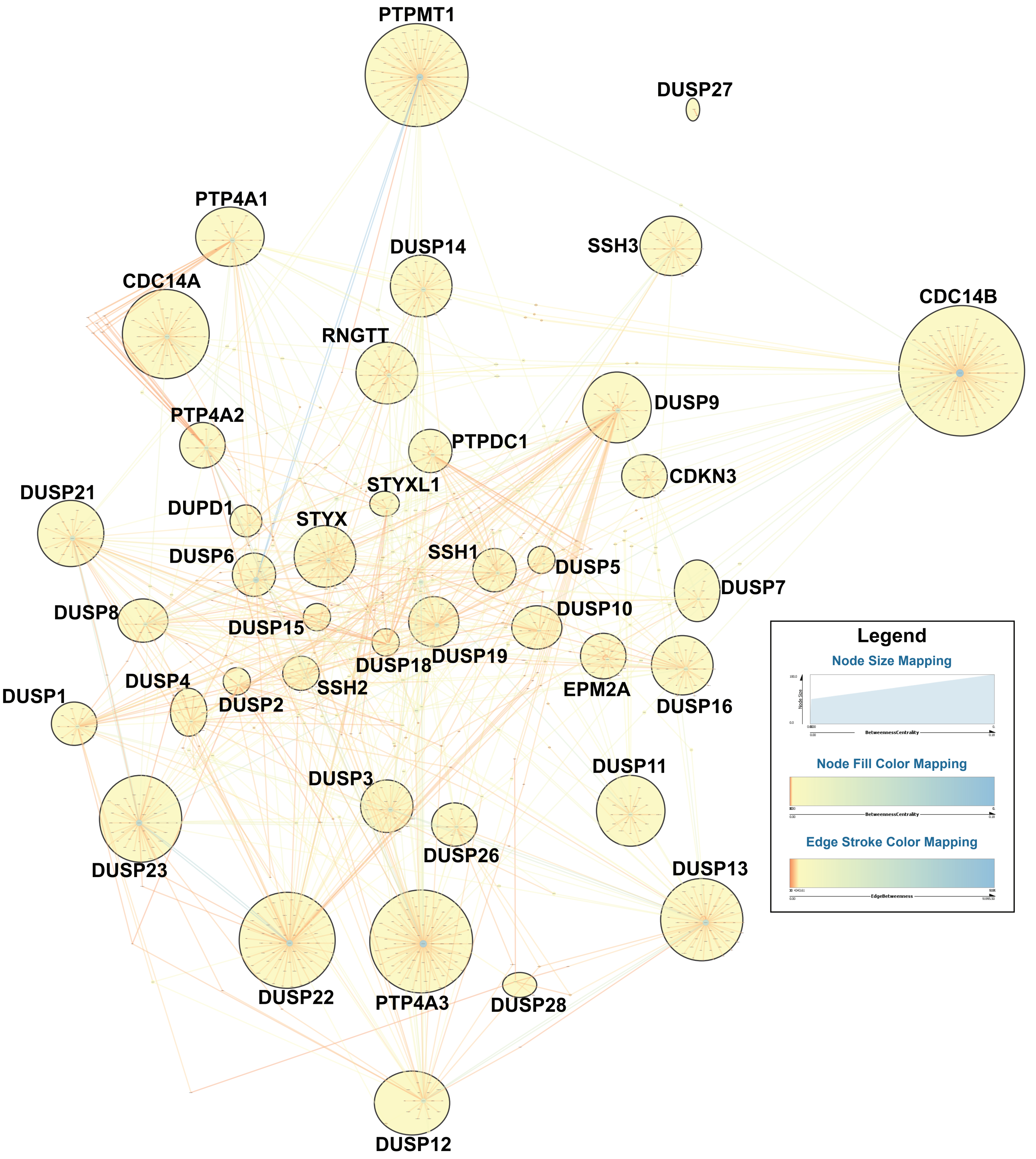

(a)

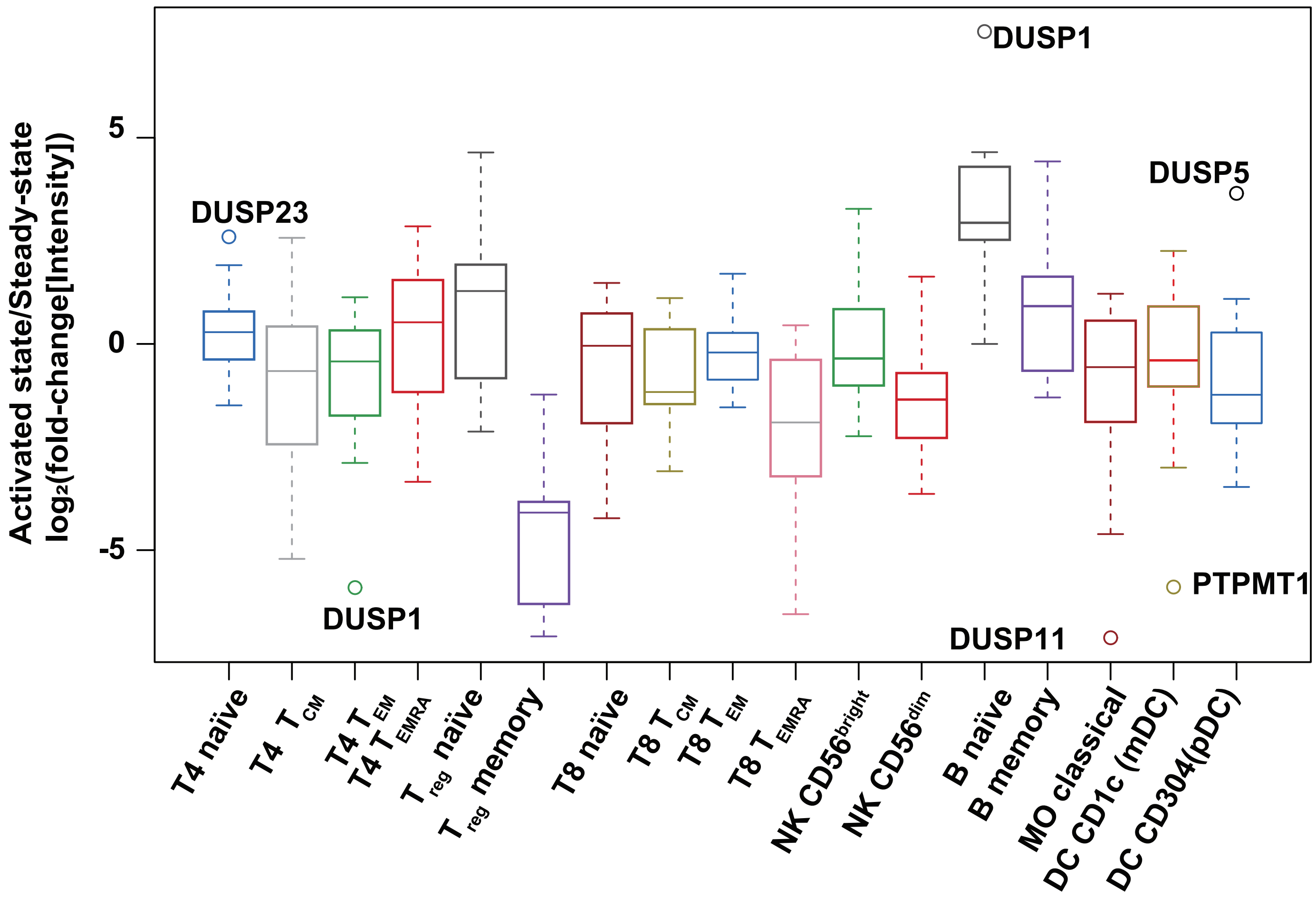
Activated state/Steady-state
log₂(fold-change[Intensity])
5
0
-5
DUSP23
DUSP1
DUSP1
DUSP5
PTPMT1
DUSP11
T4 naïve
T4 TCM
T4 TEM
T4 TEMRA
Treg naive
Treg memory
T8 naïve
T8 TCM
T8 TEM
T8 TEMRA
NK CD56bright
NK CD56dim
B naïve
B memory
MO classical
DC CD1c (mDC)
DC CD304(pDC)

(b)

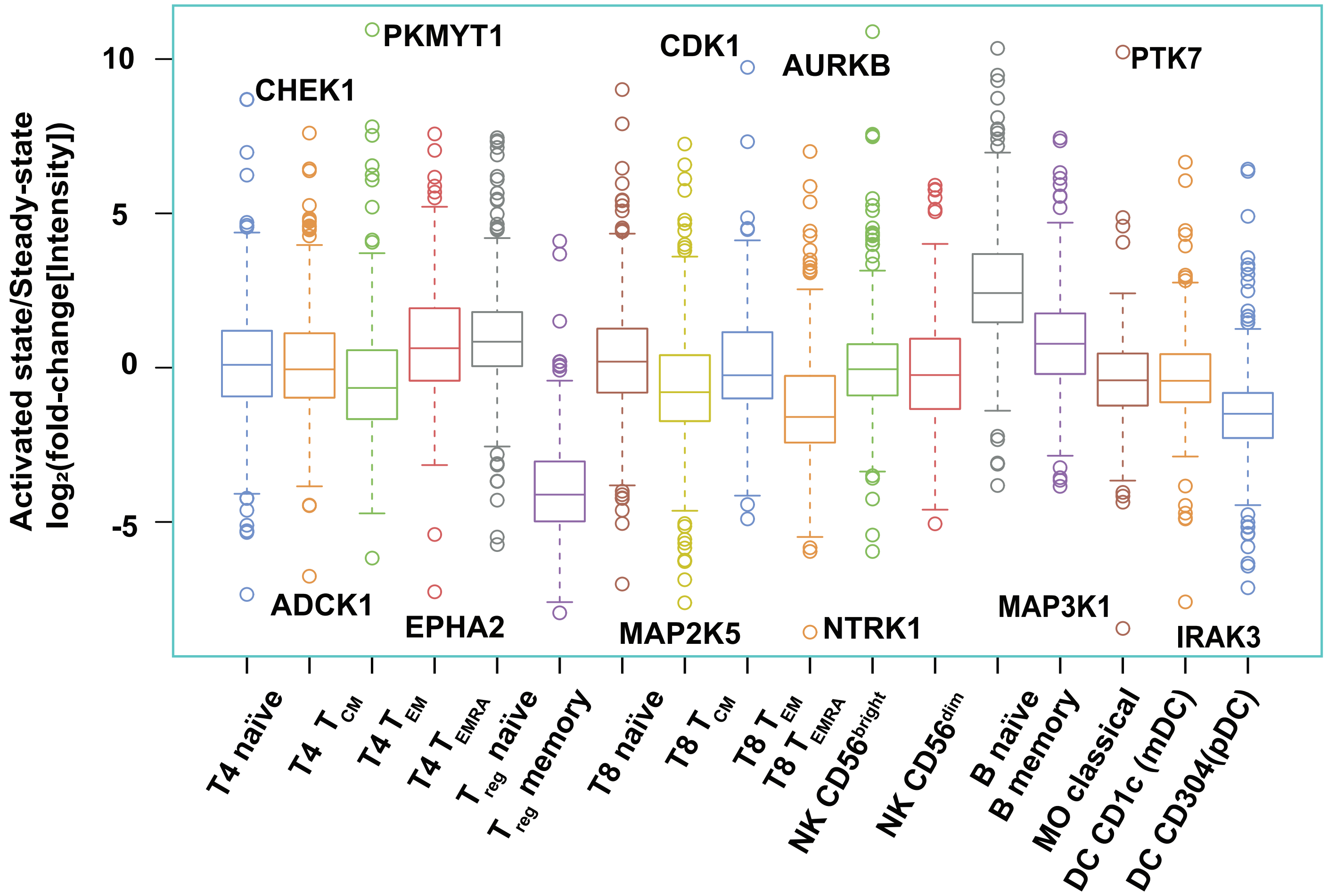
Activated state/Steady-state
log₂(fold-change[Intensity])
10
5
0
-5
PKMYT1
CHEK1
CDK1
AURKB
PTK7
ADCK1
EPHA2
MAP2K5
NTRK1
MAP3K1
IRAK3
T4 naïve
T4 TCM
T4 TEM
T4 TEMRA
Treg naive
Treg memory
T8 naïve
T8 TCM
T8 TEM
T8 TEMRA
NK CD56bright
NK CD56dim
B naïve
B memory
MO classical
DC CD1c (mDC)
DC CD304(pDC)

Supplementary figure 4

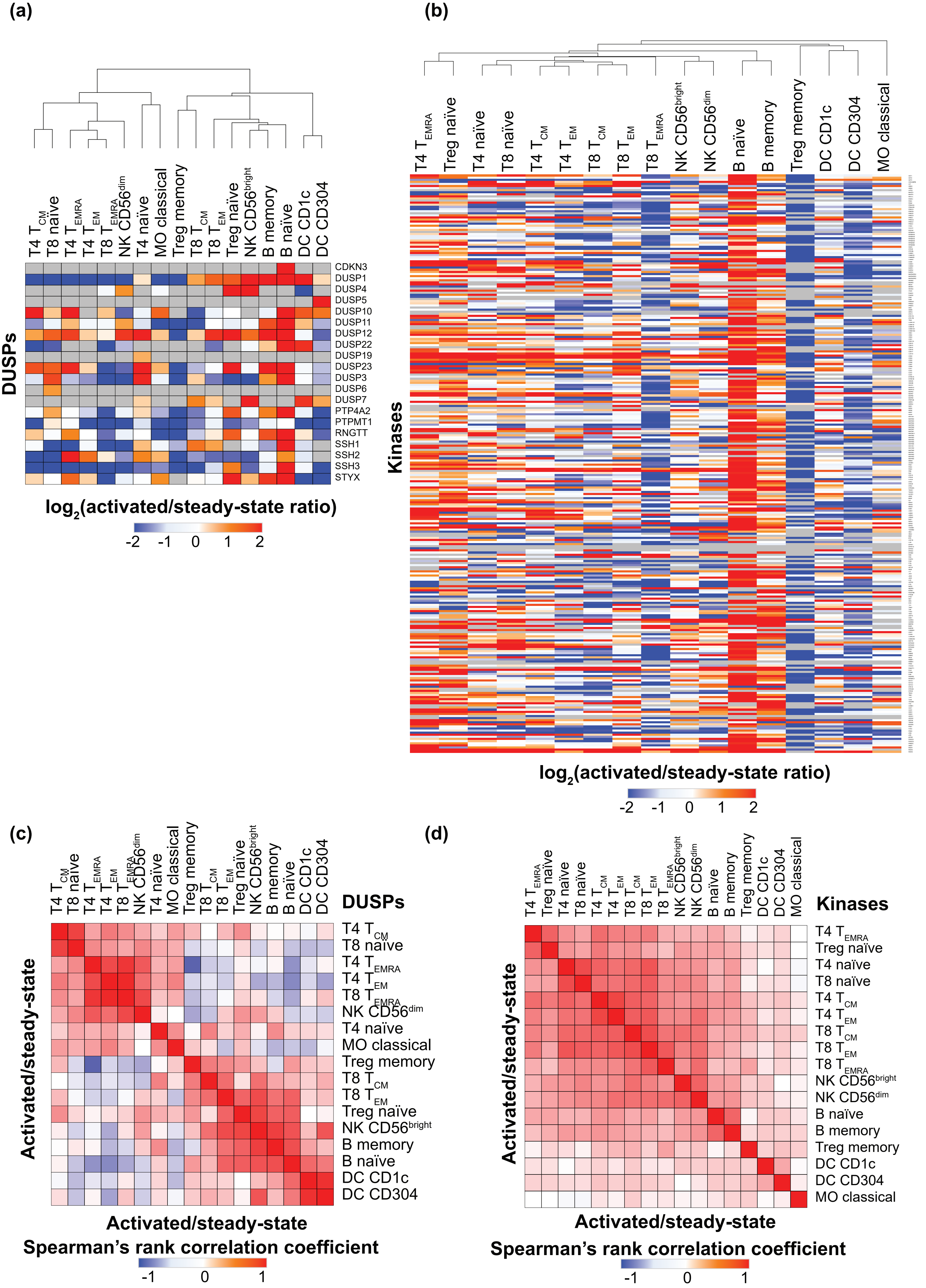

Supplementary figure 5

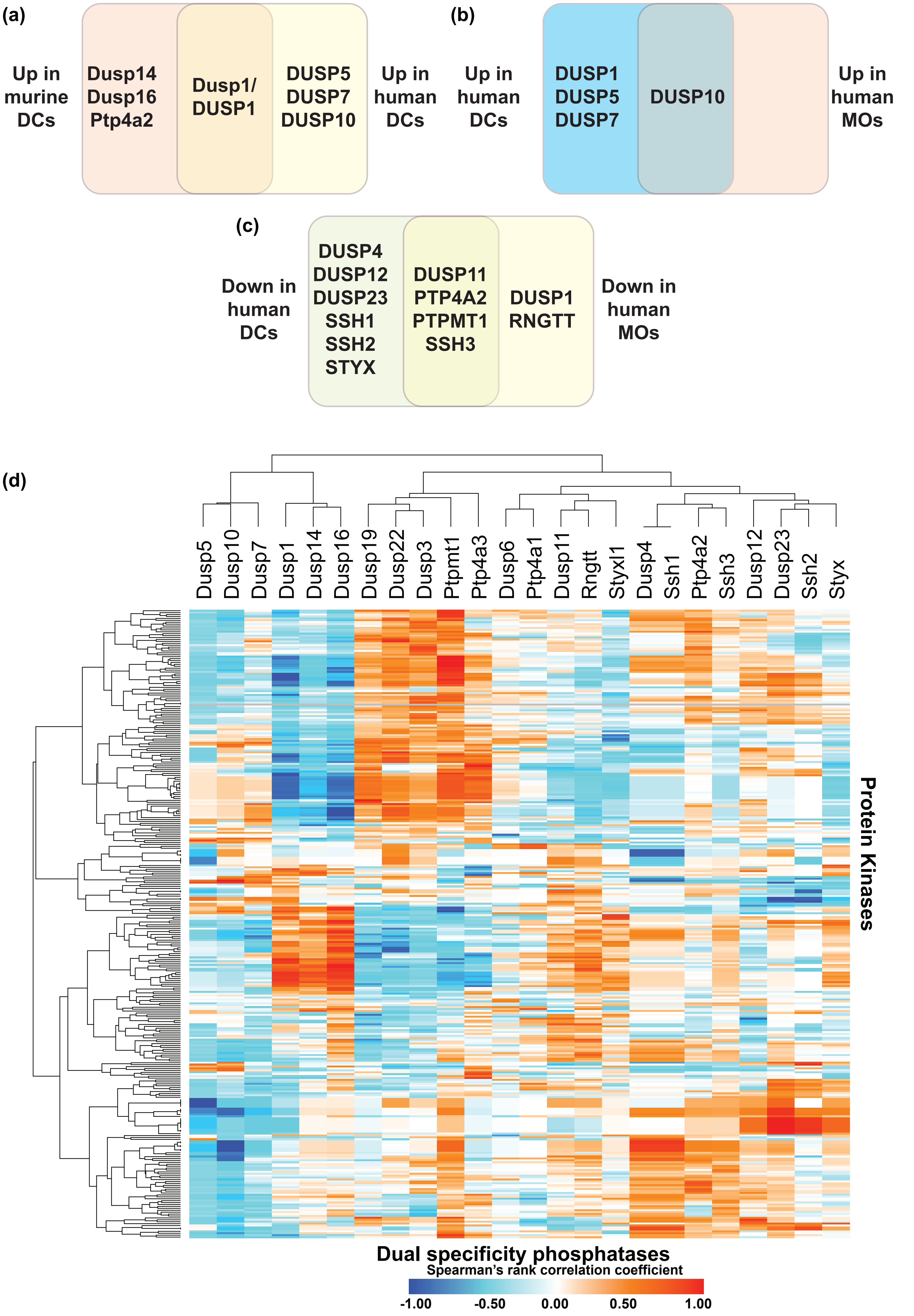

Supplementary figure 6

(a)

Upregulated molecules in murine dendritic cells

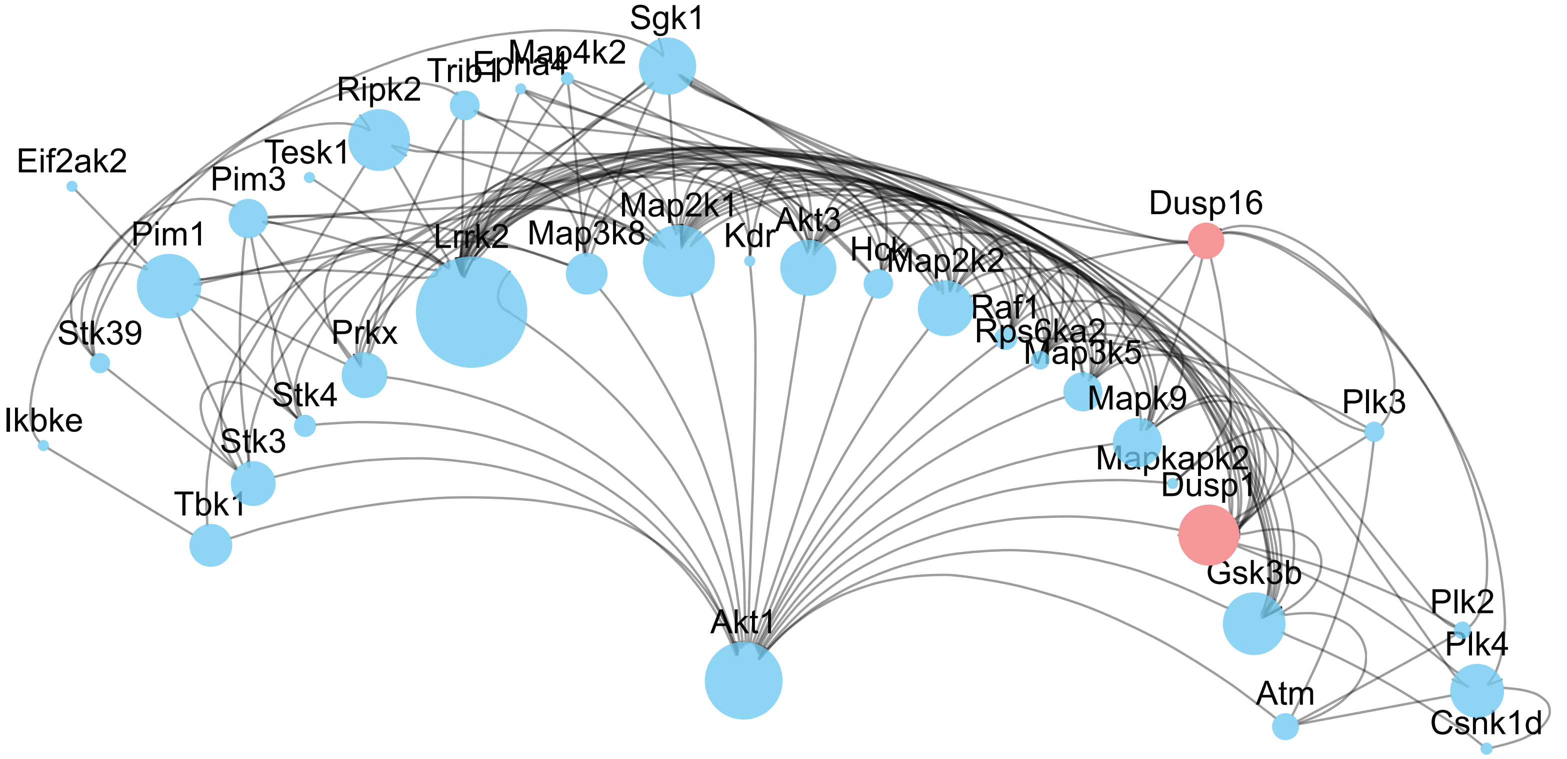

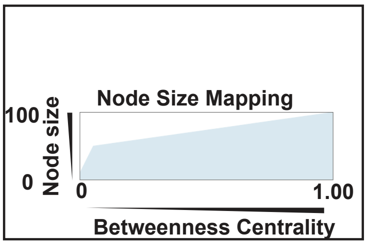

(b)

Downregulated molecules in murine dendritic cells

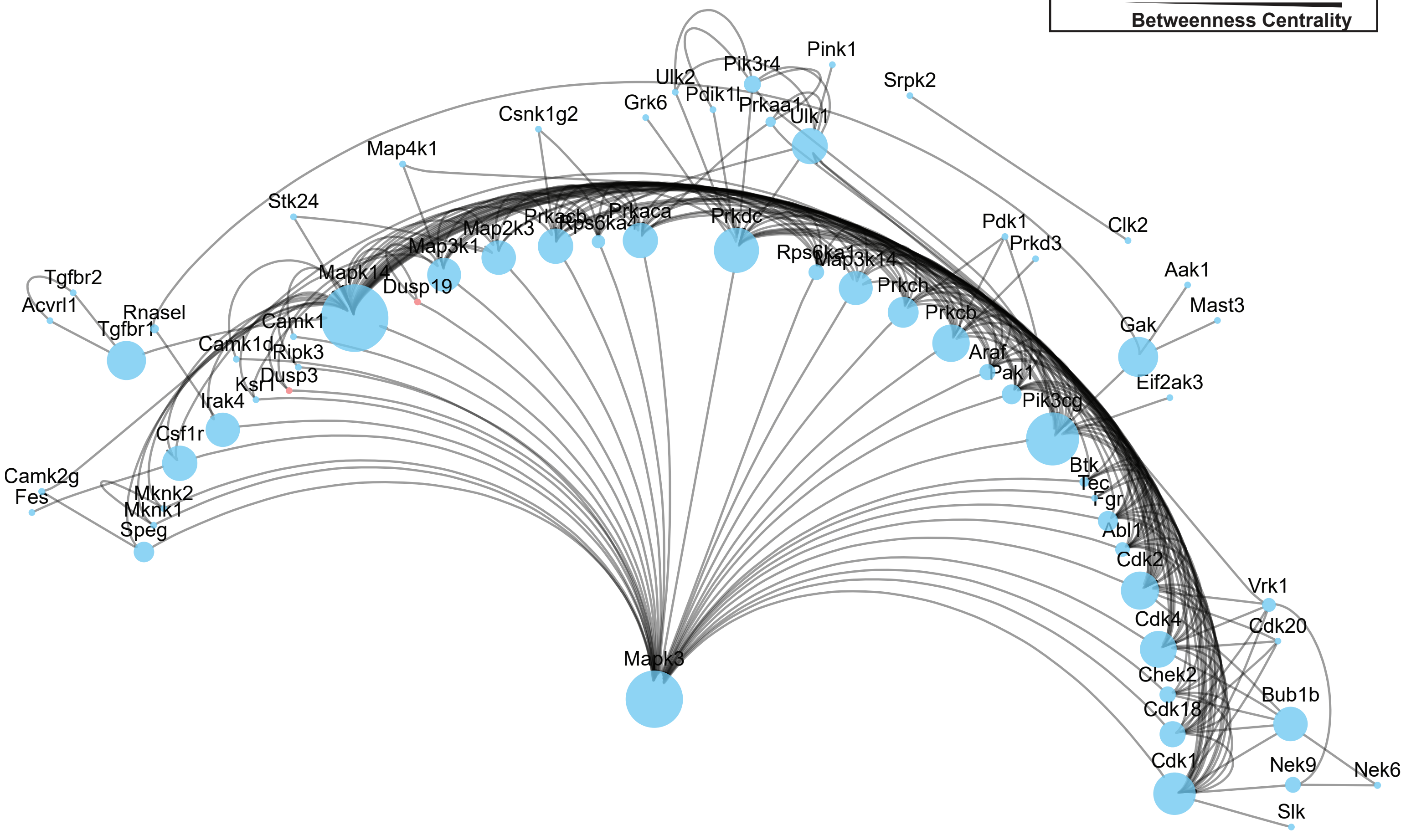

Supplementary figure 7

(a)

Upregulated molecules in human dendritic cells

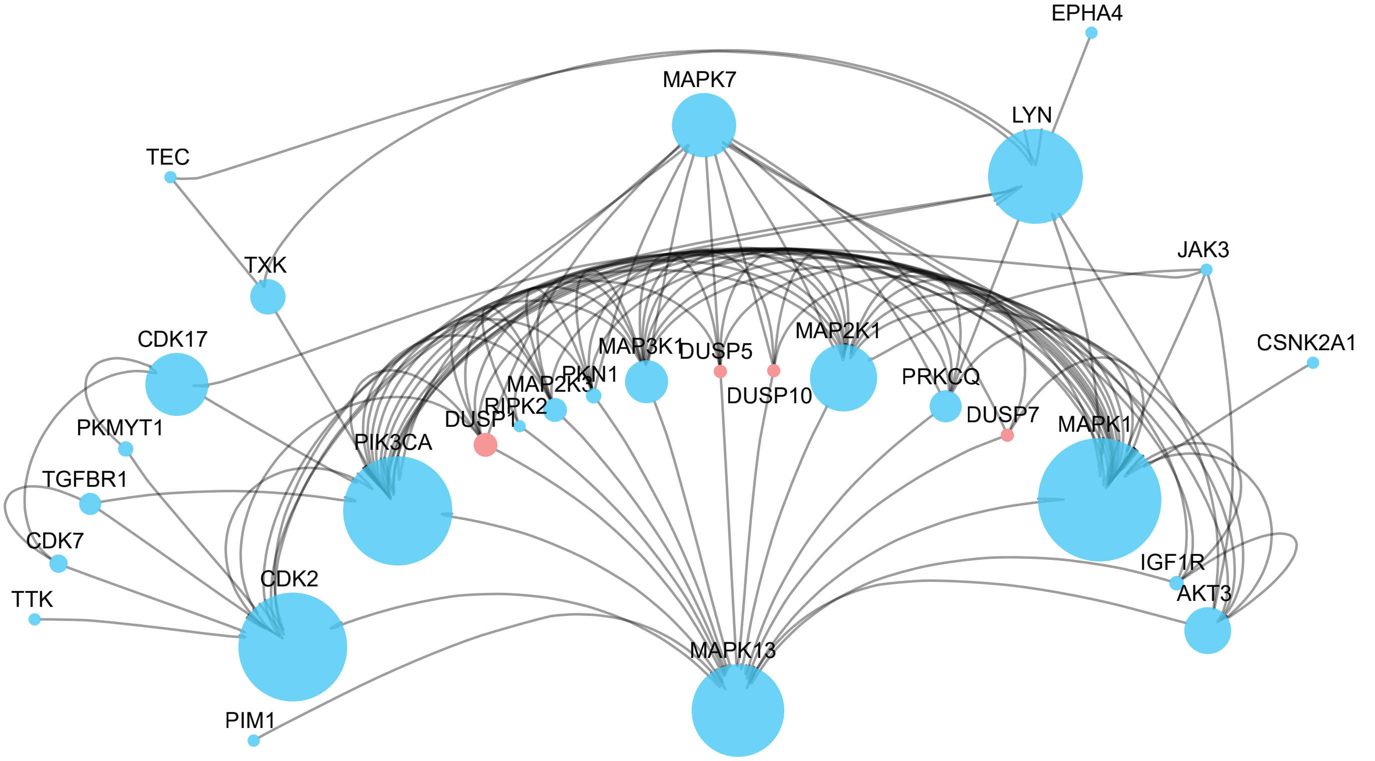

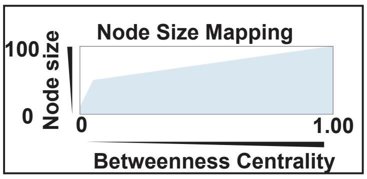

(b)

Downregulated molecules in human dendritic cells

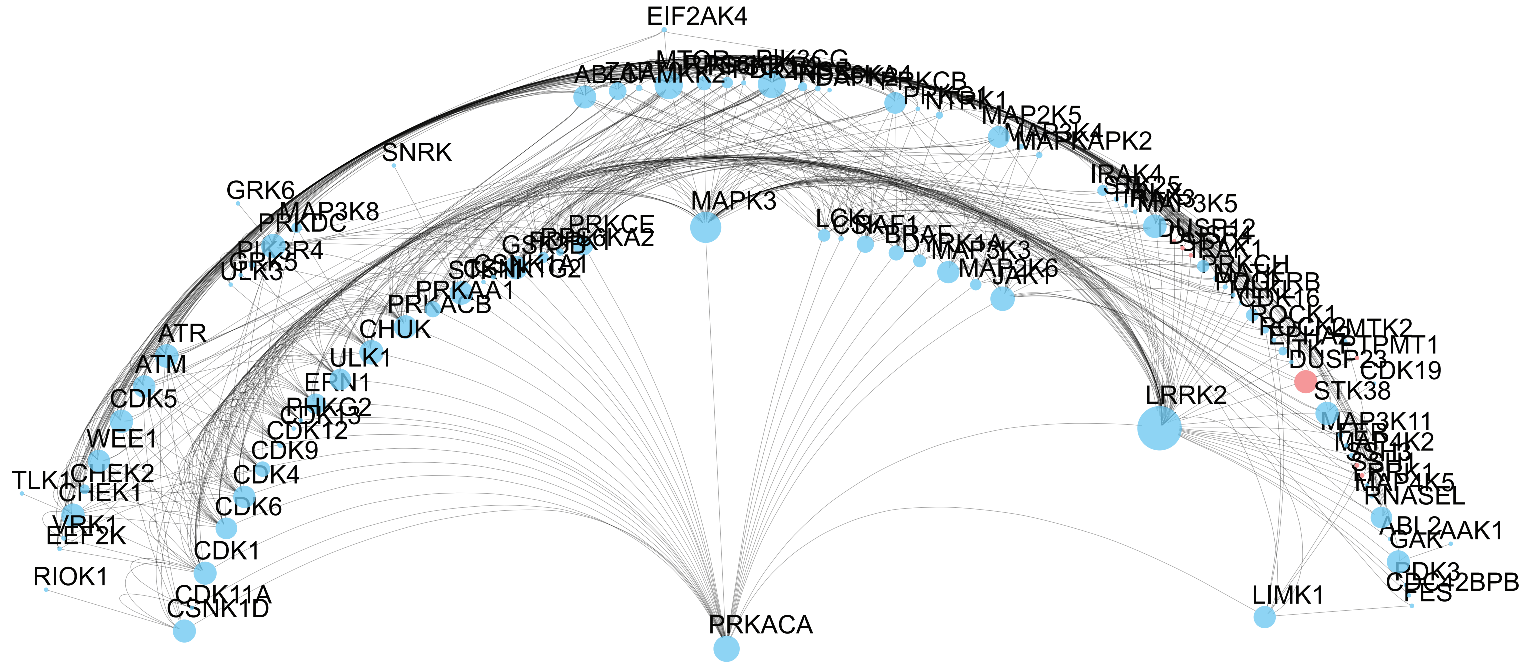

Supplementary figure 8

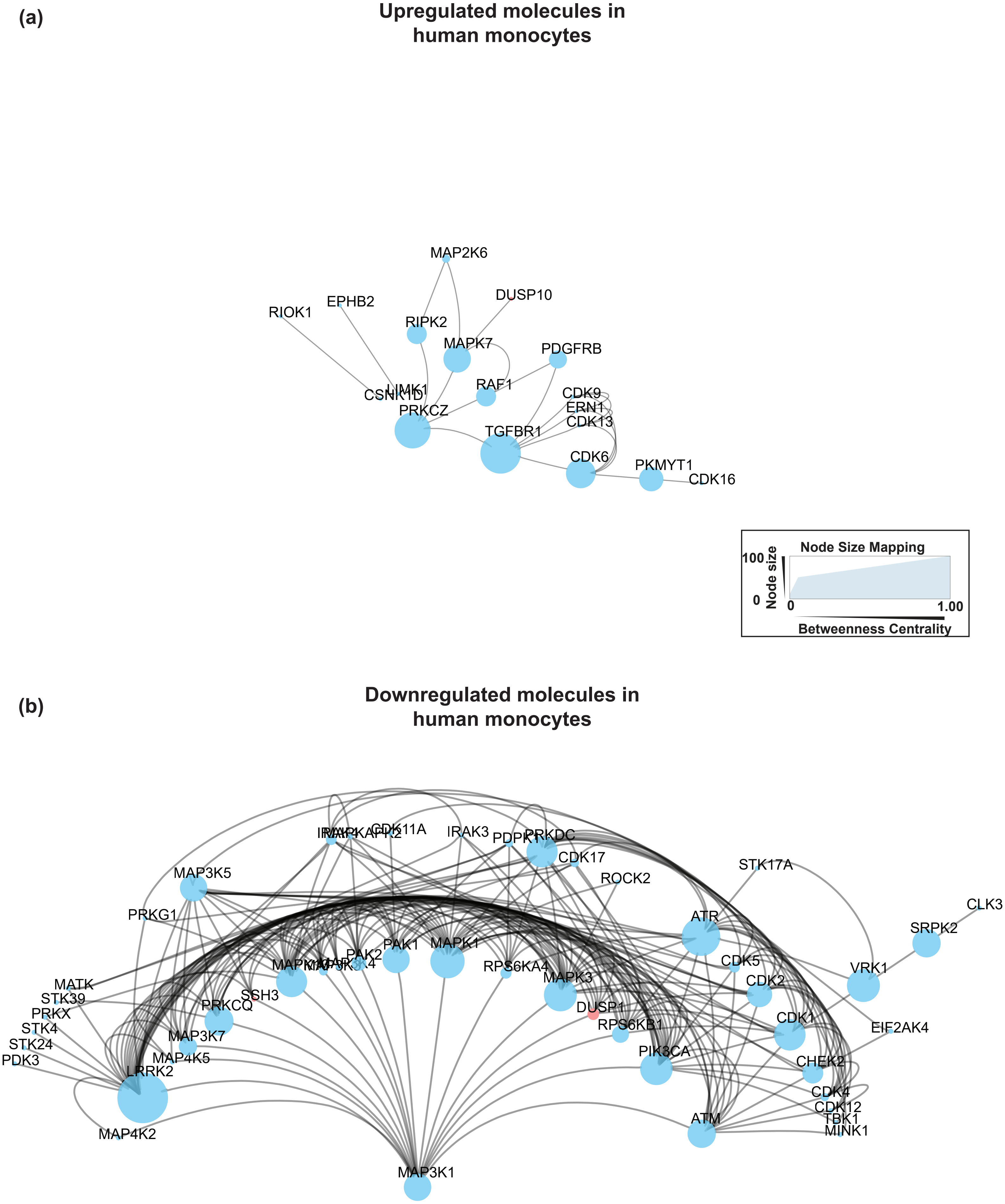

Supplement: Supplementary file 1 [file ijms-20-02086-s001.zip › Supplementary_figures.pdf]
